# From a large-scale genomic analysis of insertion sequences to insights into their regulatory roles in prokaryotes

**DOI:** 10.1186/s12864-022-08678-3

**Published:** 2022-06-20

**Authors:** Sebastien Tempel, Justin Bedo, Emmanuel Talla

**Affiliations:** 1 Aix Marseille University, CNRS, LCB, Laboratoire de Chimie Bactérienne, 13009 Marseille, France; 2 Bioinformatics Division, the Walter and Eliza Hall Institute, 1G Royal Parade, Parkville, VIC 3052 Australia; 3 School of Computing and Information Systems, the University of Melbourne, Parkville, VIC 3010 Australia

**Keywords:** Insertion sequence, IS regulatory role, IS neighboring genes

## Abstract

**Background:**

Insertion sequences (ISs) are mobile repeat sequences and most of them can copy themselves to new host genome locations, leading to genome plasticity and gene regulation in prokaryotes. In this study, we present functional and evolutionary relationships between IS and neighboring genes in a large-scale comparative genomic analysis.

**Results:**

IS families were located in all prokaryotic phyla, with preferential occurrence of IS*3*, IS*4*, IS*481*, and IS*5* families in Alpha-, Beta-, and Gammaproteobacteria, Actinobacteria and Firmicutes as well as in eukaryote host-associated organisms and autotrophic opportunistic pathogens. We defined the concept of the IS-Gene couple (IG), which allowed to highlight the functional and regulatory impacts of an IS on the closest gene. Genes involved in transcriptional regulation and transport activities were found overrepresented in IG. In particular, major facilitator superfamily (MFS) transporters, ATP-binding proteins and transposases raised as favorite neighboring gene functions of IS hotspots. Then, evolutionary conserved IS-Gene sets across taxonomic lineages enabled the classification of IS-gene couples into phylum, class-to-genus, and species syntenic IS-Gene couples. The IS*5*, IS*21*, IS*4*, IS*607*, IS*91*, IS*L3* and IS*200* families displayed two to four times more ISs in the phylum and/or class-to-genus syntenic IGs compared to other IS families. This indicates that those families were probably inserted earlier than others and then subjected to horizontal transfer, transposition and deletion events over time. In phylum syntenic IG category, Betaproteobacteria, Crenarchaeota, Calditrichae, Planctomycetes, Acidithiobacillia and Cyanobacteria phyla act as IS reservoirs for other phyla, and neighboring gene functions are mostly related to transcriptional regulators. Comparison of IS occurrences with predicted regulatory motifs led to ~ 26.5% of motif-containing ISs with 2 motifs per IS in average. These results, concomitantly with short IS-Gene distances, suggest that those ISs would interfere with the expression of neighboring genes and thus form strong candidates for an adaptive pairing.

**Conclusions:**

All together, our large-scale study provide new insights into the IS genetic context and strongly suggest their regulatory roles.

**Supplementary Information:**

The online version contains supplementary material available at 10.1186/s12864-022-08678-3.

## Background

Insertion sequences (IS) are mobile DNA repeats present in prokaryotic species [1, 2]. They can copy and move themselves into other locations of the host genome thanks to transposases. ISs are classified into families based on transposition mechanisms, transposase protein sequence(s) and terminal inverted repeat sequences [3]. IS insertions can create mutations that have negative effects on the host [4], but these insertions can also positively contribute to host adaptation [5, 6] or having a regulatory role on the neighboring gene [7–9]. Indeed, IS insertion close to a gene may create transcriptional gene regulation, such as transcription terminators, transcription factor binding sites (TFBSs) and posttranscriptional gene regulation involving small RNAs (sRNAs) [10]. Numerous studies have also shown that an IS can play a role as a promoter for neighboring genes [11–13] in a large diversity of organisms, including Enterobacteria, Bacilli and Paracoccus species [14–17]. As examples, IS*981* (from *Lactococcus lactis*) [18] and IS*903* (from the IS*5* family in Paracoccus species) were shown to drive the transcription of reporter genes in *Escherichia coli* [19]. In addition, an IS*5* insertion upstream of a promoter modifies the regulation of neighboring genes located in the ybeJ-gltJKL-ybeK operon [16] and flhDC operon [15] in *E. coli*. Finally, two TFBSs and a promoter located inside IS*1667* sequences regulate the *invA* gene in *Yersinia enterocolitica* strains [17]. All these examples highlight the significance and biological importance of IS insertions on their neighboring genes. However, no global analysis of IS functional impacts for neighboring genes or their regulatory role in gene expression was performed.

ISfinder (www-is.biotoul.fr/) is the largest IS database and provides an IS repository including almost 5000 individual IS sequences from both bacteria and archaea as well as their classification [20]. Each IS is indexed in ISfinder with various information (name, size, complete nucleotide sequence, sequences of ends and target sites, potential protein sequences, strain origin, distribution in other strains and available bibliography) and classified into families with some insights into the transposition mechanisms. The corresponding web tool ISsaga (http://issaga.biotoul.fr/ISsaga/issaga_index.php) provides general prediction and annotation tools, information on the genome context of individual ISs and a graphical overview of IS distribution within the genome of interest [21]. However, the number of prokaryotic species in ISfinder represents only a small proportion of those available in public databases with limited information on IS regulatory roles.

In this work, we undertook a large-scale genome IS survey within prokaryotic organisms, first focusing on their occurrences among the 29 IS reference families, their distribution along the genomes and their taxonomic distribution over the taxonomic lineages. Then, the concept of an IS-Gene couple (IG) was defined to explore the association of ISs with their two neighboring genes through gene orientations, gene distances, and gene functions. Comparative analysis of the IGs based on their taxonomic level as well as cross-comparison of ISs against predicted and experimentally known regulatory motifs allowed us to to reinforce IS potential regulatory roles on a large scale.

## Results

### Overview of IS occurrences in prokaryotic genomes

**Fig. 1:**
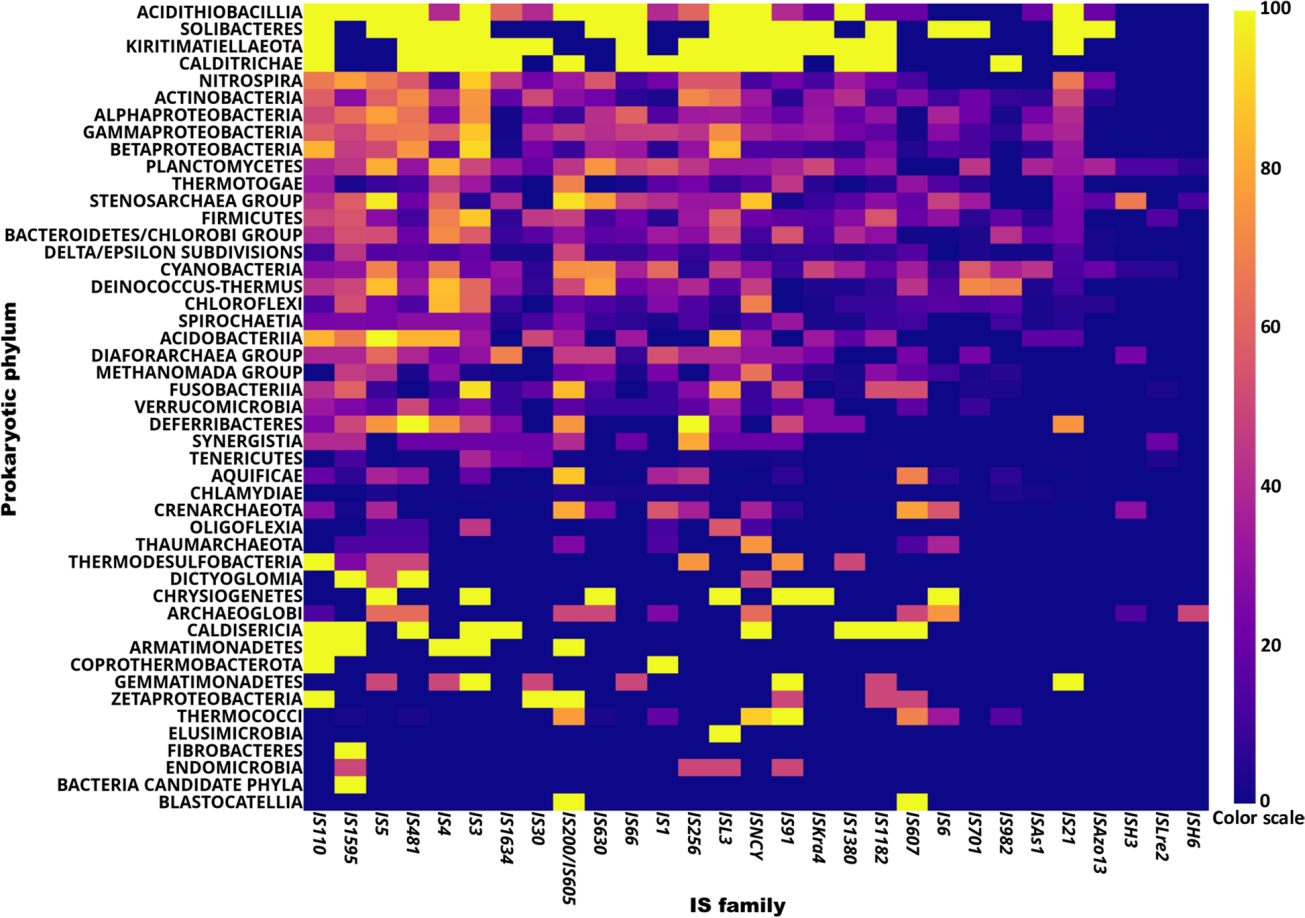
Heatmap of the IS-containing genomes among IS families and taxonomic clades. For each combination of taxonomic clade and IS family, the bar color scale corresponds to IS-containing genomes of the clade over the total number of genomes (expressed in percentage) in the same clade

IS identification resulted in 612,700 non-overlapping IS occurrences distributed on 14,151 chromosomes and plasmids located within 8481 distinct genomes (Additional file 1: Table S1). There was no correlation between the number of ISs in the genome and the genomic features, such as genome size or the number of genes (data not shown), as found by Touchon and Rocha [22]. ISs are known to use intercellular ‘mobile vehicles’ such as plasmids [4] to invade a host genome, and in agreement with this, we observed seven-fold more ISs located in plasmids than in chromosomes: one IS per 29,251 bp and 206,620 bp in plasmids and chromosomes, respectively. The proportion of IS-containing genomes within Archaea and Bacteria were 96.8 and 93.7% of their genome data, respectively. Each phylum displayed more than 57.1% of IS-containing genomes, except for Chlamydiae with only 5.7%; and all IS families were located within the 47 prokaryotic phyla but with a non-uniform distribution (Additional file 1: Table S1, S2). Indeed, when considering the number of IS-containing genomes in each IS family, there is a large variation between IS families and prokaryotic phyla with up to 2404 IS-containing genomes for IS3 in Gammaproteobacteria. Then the number of IS-containing genomes over the total number of genomes of the clade was calculated for each combination of IS family and taxonomic clade. This analysis confirmed the large distribution of IS-containing genomes among all phyla, with three main categories: the ones with < 30% IS-containing genomes (e.g., Fibrobacteres and Elusimicrobia phyla); between 30 and 80% (e.g., Actinobacteria and Planctomyces phyla); and > 80% IS-containing genomes (e.g., Acidithiobacillia) in the corresponding phylum (Fig. 1). It should be noteworthy that the number of analyzed genomes remains very low (< 9 genomes) in the clades with < 30% or > 80% IS-containing genomes. From the IS family point of view, IS*3*, IS*4*, IS*5*, IS*91*, IS*110*, IS*200*, IS*481*, ISL*3*, IS*1595*, and IS*NCY* were located in at least 30 distinct phyla (with a maximum of 35) while IS*H3*, IS*H6* and IS*Lre2* were found in less than 9 distinct phyla. In the next step, statistical analysis displayed preferential insertions (*p value* < 0.05) for IS*As1*, IS*607*, IS*701*, IS*982*, IS*1634*, IS*Azo13*, IS*Lre2*, IS*H3*, and IS*H6* families (Additional file 1: Table S2). Indeed, in Firmicutes, the IS*607* and IS*982* families also have a strong preferential insertion in 371 and 340 genomes, while predictive insertions from the uniform distribution should be 207 and 175 genomes, respectively. The IS*Lre2* family is almost exclusively present in the Firmicutes phylum (266 genomes; 92.0% of the total IS*Lre2-*containing genomes) but is only found in 23 genomes of other phyla. Indeed, IS*Lre2* is a small family (49 IS members in ISfinder) for which no IS distribution study along complete genomes is available. These observations provide some clues about preferential insertions of IS*Lre2* in Firmicutes and therefore link this IS family to specific lifestyle environments of Firmicutes or specific host factors for their transposition in the clade. Other preferential IS insertions related to specific phyla were found, as follows: the IS*Azo13* family was mainly identified in 54 (40.2% of IS*Azo13*-containing genomes) actinobacterial genomes (representing 10.6% of the overall genomes) but located in 3 (2.2% of IS*Azo13*-containing genomes) gammaproteobacterial genomes (representing 30.2% of the overall genomes) and 77 genomes (57.6% of IS*Azo13*-containing genomes) of the remaining phyla (representing ~ 60% of the overall genomes). IS*1634* also has preferential insertion in Actinobacteria with 170 genomes, while a uniform distribution predicts only 60 genomes. Finally, IS*H3* and IS*H6* have very strong preferential insertions in archaeal genomes, particularly within the Stenosarchaeal group, with ISs located in 59 and 10 genomes, respectively. Moreover, the 15 IS*H6*-containing genomes (e.g., *Halobacterium salinarum* NRC-1 and *Archaeoglobus fulgidus* DSM 8774) share common lifestyles (lake and sea environments with very high salt concentrations), suggesting that IS*H6* occurrences could be limited to a specific environment and could be used as a genetic marker for organisms growing in habitats with high salt concentrations.

**Fig. 2:**
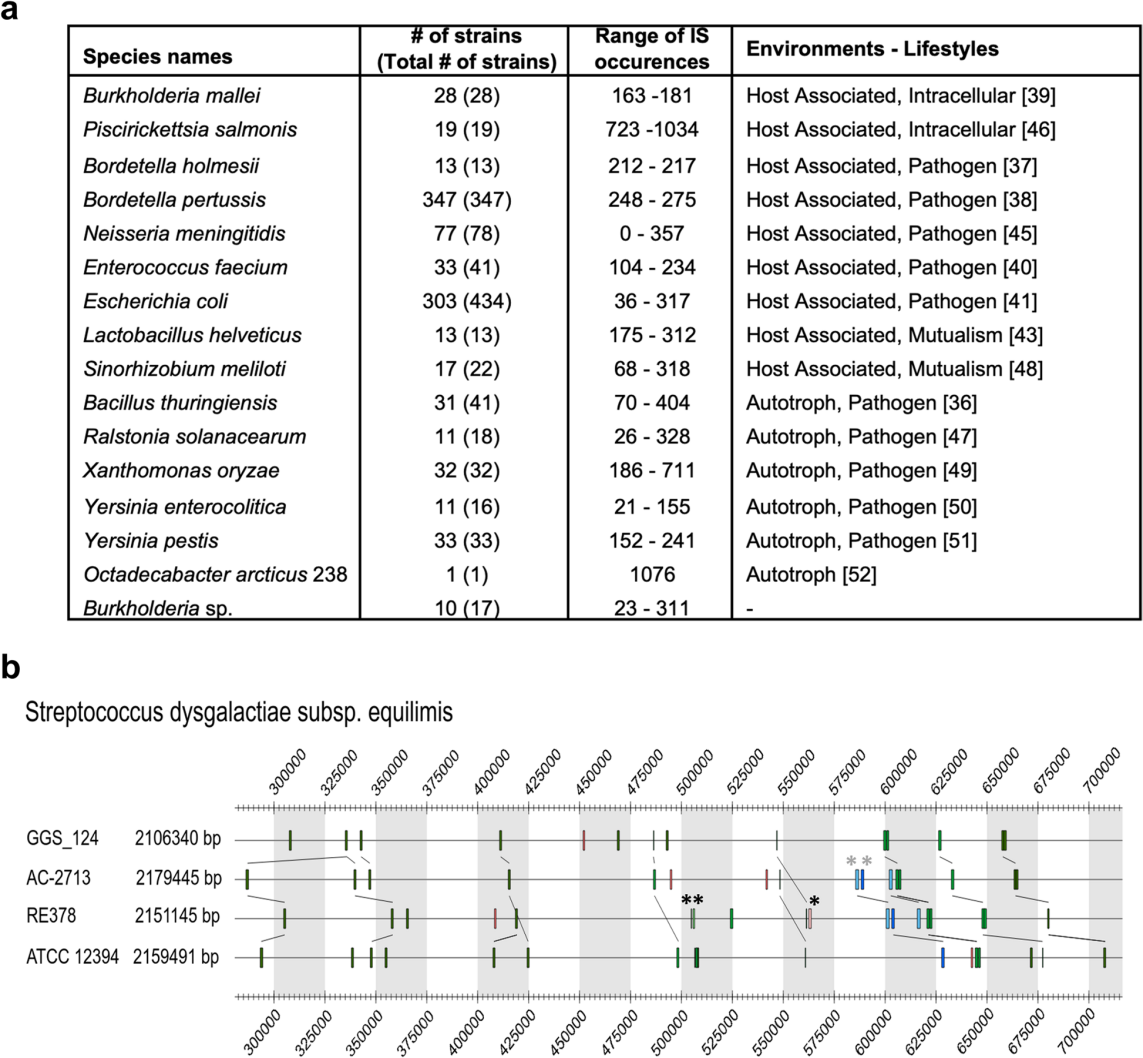
**a**. Genomes with the highest number of IS occurences. The chosen species are species that have at least 100 IS occurrences. The number of strains with at least 100 IS occurences is shown with the total number of strains in paranthesis, followed by the range of IS occurrences and the environment and lifestyle associated to species. **b**. Variability of IS occurrences within strains of the same species: the case of *Streptococcus dysgalactiae subsp. Equisimilis* species. The strain name, genome size as well as part of the genomic map of the strains are shown. Each colored bar corresponds to an IS occurrence of a given IS superfamily. In this region, same IS families between two strains are connected with lines. The strain RE378 contains two specific IS families (IS*256* and IS*As1*) (when compared to the 3 other strains) are marked with black *, while two IS families (IS*110* and IS*1182*) (with grey *) are absent from the strain GGS_124

Data analysis also revealed that 20.3% (1839 genomes) of the overall genomes exhibited at least 100 identified IS occurrences each, with 13 genomes possessing at least 1000 IS occurrences (Additional file 1: Table S1). These genomes are mainly in the Actinobacteria (105 organisms), Alphaproteobacteria (139), Betaproteobacteria (583), Firmicutes (251) and Gammaproteobacteria (603) phyla. *Octadecabacter arcticus* 238 (Alphaproteobacteria, GCA_000155735.2) [23], which lives in the Arctic Sea, is the genome containing the highest number of ISs: 1076 IS sequences (spanning ~ 21.02% coverage size of the total genome) distributed in 20 distinct IS families, among which the IS*3* family (with 342 IS occurrences) remains the most important family. Analysis of an organism’s lifestyle indicated that there was no specific habitat, temperature range or disease associated with these 1839 “high IS content” organisms. However, a subset of 16 species (in which at least 50% of strains contain a minimum of 100 IS occurrences per genome) (Fig. 2a) showed that they interact with eukaryotic organisms and fall into the following two categories: host-associated (which corresponds to prokaryotes that cannot live without the interaction with eukaryotes) and autotrophic opportunistic pathogens (which are able to produce their own energy source). In addition, the ‘host-associated’ lifestyle was also found related to mutualism with *Lactobacillus helveticus* and *Sinorhizobium meliloti* and intracellular pathogenicity with *Piscirickettsia salmonis* and *Burkholderia mallei*. Finally, *Bordetella holmesii*, *Bordetella pertussis*, *Enterococcus faecium*, *Escherichia coli*, *Neisseria gonorrheae*, and *Neisseria meningitidis* could cause diseases. The second category (autotrophs and pathogens) corresponds to prokaryotes that live in terrestrial or aquatic environments but could create diseases when they interact with eukaryotes, such as *Bacillus thuringiensis*, *Ralstonia solanacearum*, *Xanthomonas oryzae*, *Yersinia enterocolitica* and *Yersinia pestis*. These results suggest that the high number of IS copies can help organisms to adapt to distinct lifestyle environments.

As recently shown for *Micrococcus luteus* strains [24], Fig. 2a (and Additional file 1: Table S3) also highlighted the large variations in IS occurrences within strains of the same species. As examples, the number of IS occurrences in the 19 strains of *Piscirickettsia salmonis* and 434 strains of *Escherichia coli* species varies from 723 (*Piscirickettsia salmonis* PM15972A1, GCA_000756435.3) to 1034 (*Piscirickettsia salmonis* PM58386B, GCA_001932835.1) and 36 (*Escherichia coli* LF82, GCA_000284495.1) to 317 (*Escherichia coli* strain ECONIH5, GCA_002903105.1), respectively. In addition, one frequently observed is the location of distinct IS families in the same genomic region within strains of the same species. This could be illustrated within a genomic sequence view of *Streptococcus dysgalactiae subsp. equisimilis* organisms (Fig. 2b), for which the four strains show the location of specific IS families (e.g. IS*256* and IS*As1* in *S. dysgalactiae subsp. equisimilis* RE378). These specific IS insertions in closely related strains can be used as markers for the identification and classification of bacterial strains at the species/strain level when classical in silico methods (e.g., phylogenetic analysis) cannot.

### The concept of IS-gene couple allows us to explore the biological relationship between the IS and neighboring genes

**Fig. 3:**
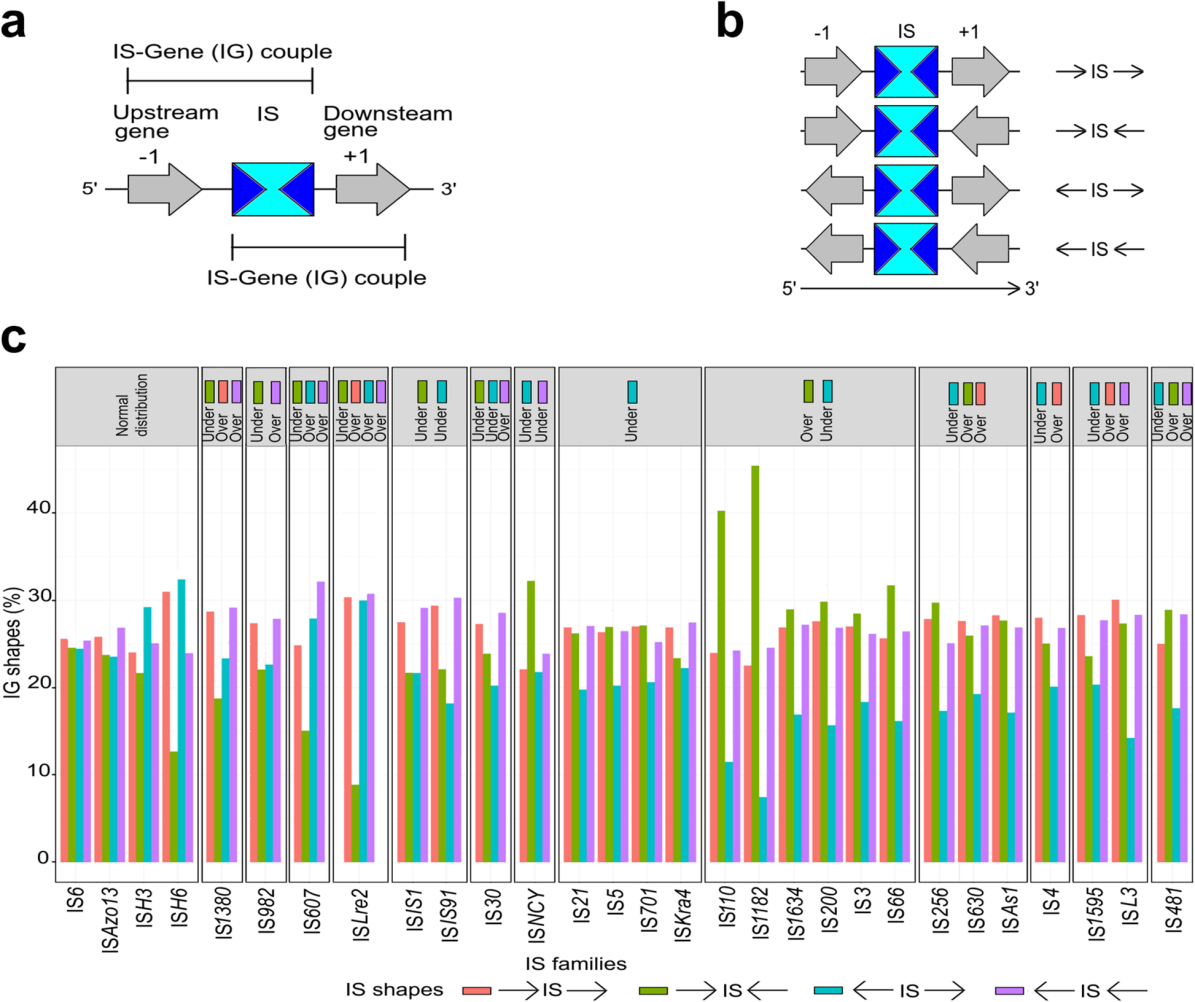
**a**. A typical IS associated to their neighbouring genes. Upstream (− 1) and downstream (+ 1) genes are relative to the genome sequence 5′-3′ orientation. The two IS-Gene couples (IG) for (− 1) or (+ 1) gene are indicated. When IS overlap a gene, the latter was denoted as 0. **b**. Types of IG shapes between IS and neighbouring genes. Arrows indicate the orientation of − 1 (downstream) and + 1 (upstream) genes. The four distinct IG shapes are: →IS→, Same orientation on Positive Strand; ←IS←, Same orientation on Negative Strand; →IS←, Opposite and Convergent orientation; ←IS→, Opposite and Divergent orientation. **c**. Distribution of IS-Gene (IG) shapes. When they exist, normal, underrepresentation and overrepresentation of IG shapes compared to a random distribution are displayed (with the corresponding color codes) for each IS family

Considering both gene orientations, the concept of IS-Gene couple (named IG) for each IS occurrence was defined, leading to four IG shapes as follows (Fig. 3a, b): →IS→ and ←IS←, both corresponding to an IS insertion inside (or within a promoter region of) a transcript unit or an operon or within the transcription terminal regions of neighboring genes, the →IS← shape that corresponds to the end of two operons or an intergenic region; and ←IS→ that corresponds to the IS insertion in the promoter region of the two genes (or beginning of both operons). Figure 3c (and Additional file 1: Table S4) show that the neighboring gene orientations relative to the IS insertions are variable (→IS←, 18.3% of the total IG shapes; ←IS→, 28.3%; →IS→, 26.5%; and ←IS←, 26.9%) and that the 29 IS families could be grouped into 14 categories, depending on normal, over-, or underrepresentation of the IG shapes. Several facts can be pinpointed, as follows: (*i*) the IS*6*, IS*Azo13*, IS*H3* and IS*H6* families displayed a ‘normal’ distribution for the four IG shapes, even if the I*SH6* family has few IS occurrences; (*ii*) underrepresentation of insertions (compared to what statistically expected) was mainly found in 21 IS families (e.g., IS*1*, IS*21* and IS*4*; percentage of ←IS→ shapes ranging from 7.5 to 22.3%) and 7 IS families (e.g., IS*91* and IS*607*; percentage of →IS← shapes ranging from 8.9 to 23.9%) for the ←IS→ and →IS← neighboring gene orientations, respectively; and (*iii*) overrepresentation of the IG shapes are mostly found in 4 IS families (e.g., IS*1380* and ISL*3*; percentage of insertions ranging from 27.7 to 30.8%) for both the →IS→ and ←IS← shapes. In addition, IS*607* and IS*Lre2* as well as IS*30* and IS*481* are overrepresented in the ←IS→ and ←IS← orientations, respectively. Overrepresented →IS← shapes in the IS*3*, IS*66*, IS*110*, IS*200*, IS*256*, IS*481*, IS*630*, IS*118*2, IS*1634* and IS*As1* families suggest that the 3′ end genetic region (mainly composed of IS) may play a role in gene regulation and that these IS insertions may lead to a beneficial role. Moreover, IS*1380*, IS*1595*, IS*Lre2* and IS*L3* occurrences were overrepresented between genes (in both the →IS→ and ←IS← shapes), meaning that IS occurrences should be more conserved in their host genomes if they do not modify the regulation of the gene or have a silent role.

### Overrepresented IGF gene functions are mainly related to transcriptional regulation and transport activity

A total number of 30,769,611 genes in 8481 genomes led to 117,851 distinct functional descriptions (referred here as protein-coding gene functions or gene functions) in unique and multiple copies. Among them, the most represented ‘gene functions’ were ‘hypothetical protein’, ‘ABC transporter ATP binding protein’ and ‘MFS transporter’, which accounted for 19.4% (5,947,640 genes), 0.99% (303,034) and 0.95% (291,137) of the total number of genes, respectively (Additional file 1: Table S5). When gene functions were combined with the IS families, 104,094 distinct IS-GeneFunction (IGF) couples (from a total of 1,577,486 IGF couples) could be observed, with most of them displaying a unique combination of a given IS family and gene function. For example, 2020 distinct IS*1*-GeneFunction couples (among a total of 3930 IS*1*-Gene couples) were unique (Additional file 1: Table S6). Our findings of unique and multiple copies of IGF couples clearly highlight the multiple strategies of IS invasion among prokaryotes, including IS insertion alone without any evolutionary events or IS insertion with vertical inheritance or horizontal transfer events. IGF couples in multiple copies suggest their specific conservation among distinct phyla or across evolutionary history and therefore a possible role of these ISs in their neighboring genes.

**Fig. 4:**
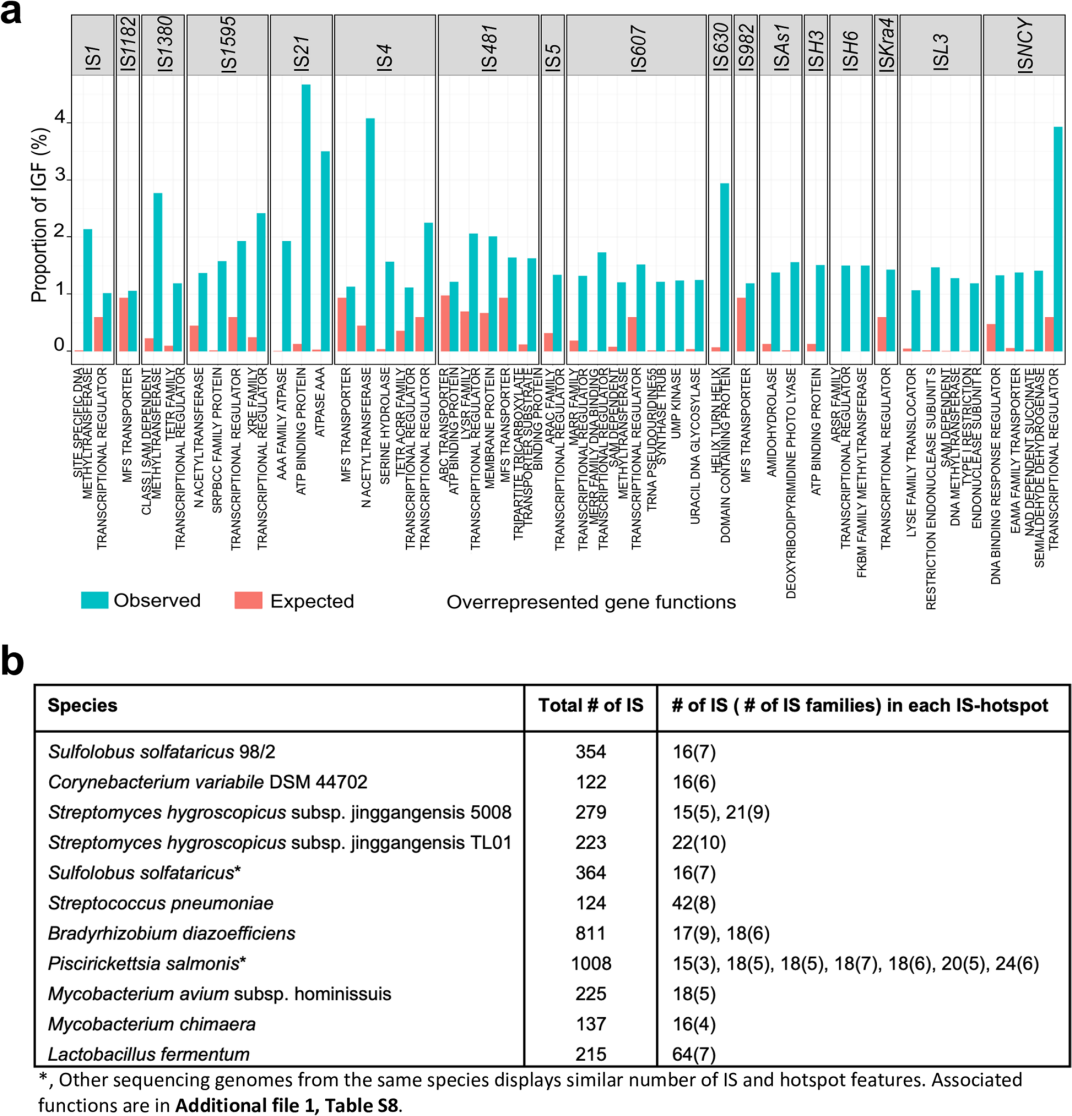
**a**. Overrepresented gene functions in IS-Gene couples among IS families. For a given IS family, overrepresented gene functions are displayed when the corresponding IS-Gene Function (IGF) represents more than 1% of the IGF couples with at least 50 IG couples (See Materials and methods). The blue (red) bar shows the observed (expected) percentage of IGFs. **b**. Selected IS hotspots. The total number of IS within the genome as well as the number of IS and distinct IS families located within the given IS-hotspot are given

Statistical analysis led to 29,663 distinct IGF couples (29.5% of the overall IGF) distributed in 15,610 (52.6%), 10,943 (36.9%), and 3110 (10.5%) normal, under- and overrepresented distributions, respectively (Additional file 1: Table S7). Except for ‘hypothetical protein’ (28.5% of the overall IGFs) and transposase/integrase/recombinase (relative to IS insertion mechanisms) (21.6% of the overall IGFs) proteins, 46 overrepresented distinct IGFs (from the 3110 IGF copies) with more than 1% of the total IGF couples of a given IS family (a total of 17 IS families involved) could be highlighted (Fig. 4a). Among the overrepresented IGFs, ‘IS*21* - ATP binding protein’, ‘IS*4* - N-acetyltransferase’ and ‘IS*NCY* - transcriptional regulator’ displayed the highest observed percentages of 4.67, 4.08 and 3.93%, respectively. It is interesting to note that among the overrepresented functions, 16 belong to the ‘transcriptional regulator’ gene group and 7 belong to the ‘transporter’ group. When examined, the ‘transcriptional regulator’ function is related to transcription regulation with HTH (helix turn helix) protein domains (e.g., MARR, ARAC and ARSR in IS*607*, IS*5* and IS*H6*, respectively). In the case of the ‘Transporter’ group, the ‘MFS TRANSPORTER’ gene function was overrepresented with IS*1182*, IS*4*, IS*481* and IS*982* and underrepresented in ten other IS families (e.g., IS*1380*) (Additional file 1: Table S7). These results indicate that gene function is an important factor that allows IS insertion/retention in genomic locations with preferential insertions close to protein-coding genes with functional descriptions related to ‘transcriptional regulation’ and ‘transporter’. However, the high number of distinct IGF couples also suggests that IS sequence insertions are able to target a variety of neighboring protein-coding gene functions. Definitively, while multiple factors including the DNA target, the host lifestyle, the host machineries, as well as the strength and the efficacy of the purifying selection [1] strongly influenced the IS insertion, our results suggest that IS insertion also relies on its specific family and neighboring gene functions.

### IS hotspots are target sites for the insertion of new ISs with favorite neighboring gene functions including major facilitator superfamily (MFS) transporters, ATP-binding proteins and transposases

IS distribution analysis (with genomes containing at least 10 IS occurrences) showed that 684 genetic objects (539 chromosomes and 145 plasmids) (representing 7.25% of the total) displayed a nonrandom IS distribution along the genomes, with a significant statistical value (*p value* < 0.01) (Additional file 1: Table S8). Moreover, IS distribution analysis was also performed for each IS family, showing that genomic locations that accumulate IS occurrences are not specific to an IS family or to a specific phylum. Interestingly, a few strains from these genomes also displayed the highest (≥ 100) IS occurrences (e.g., *Octadecabacter articus* 238, five of 19 strains of *Piscirickettsia salmonis*, one strain of *Xanthomonas oryzae* and 24 strains of *Escherichia coli*), suggesting possible IS hotspots within these genomes. Indeed, a subset of IS hotspots (as defined in Materials and Methods) with the highest number of IS occurrences is shown in Fig. 4b. Most of the IS hotspots are composed of several distinct IS families, and most of the strains with IS hotspots correspond to those with the highest number of ISs among the species. For example, in *Lactobacillus fermentum* organisms, the IS hotspot contains 64 ISs from the following seven distinct IS families: IS*256*, IS*200*/IS*605*, IS*3*, ISL*3*, IS*4*, IS*30*, and IS*982*. The first two families display the majority of ISs of this hotspot (12 and 24 IS, respectively). These results confirm on a large genomic scale that IS hotspots are target sites for new IS insertions, as observed [25], and that ISs and other mobile elements can drive rearrangements in prokaryotic genomes [26, 27]. Gene functions associated with these IS hotspots were also explored (Additional file 1: Table S9) and showed that among the 971 IS hotspots, 171 were located (at least two times) close to the same gene function (‘hypothetical protein’ excluded) (e.g., 9 hotspots were close to ‘LysR family transcriptional regulator’ function gene). Among the favorite IS hotspots neighboring gene functions, the ‘MFS transporter’, ‘ATP-binding protein’ and ‘transposase’ gene functions were observed. The latter gene function was not a surprise since transposition mechanisms of insertion involve transposase proteins. Altogether, our results suggest that IS hotspot creation is not specific to a particular IS family and that some genes tolerate more ISs in their genetic environment than others.

### General features associated with syntenic IS-gene couples

**Fig. 5:**
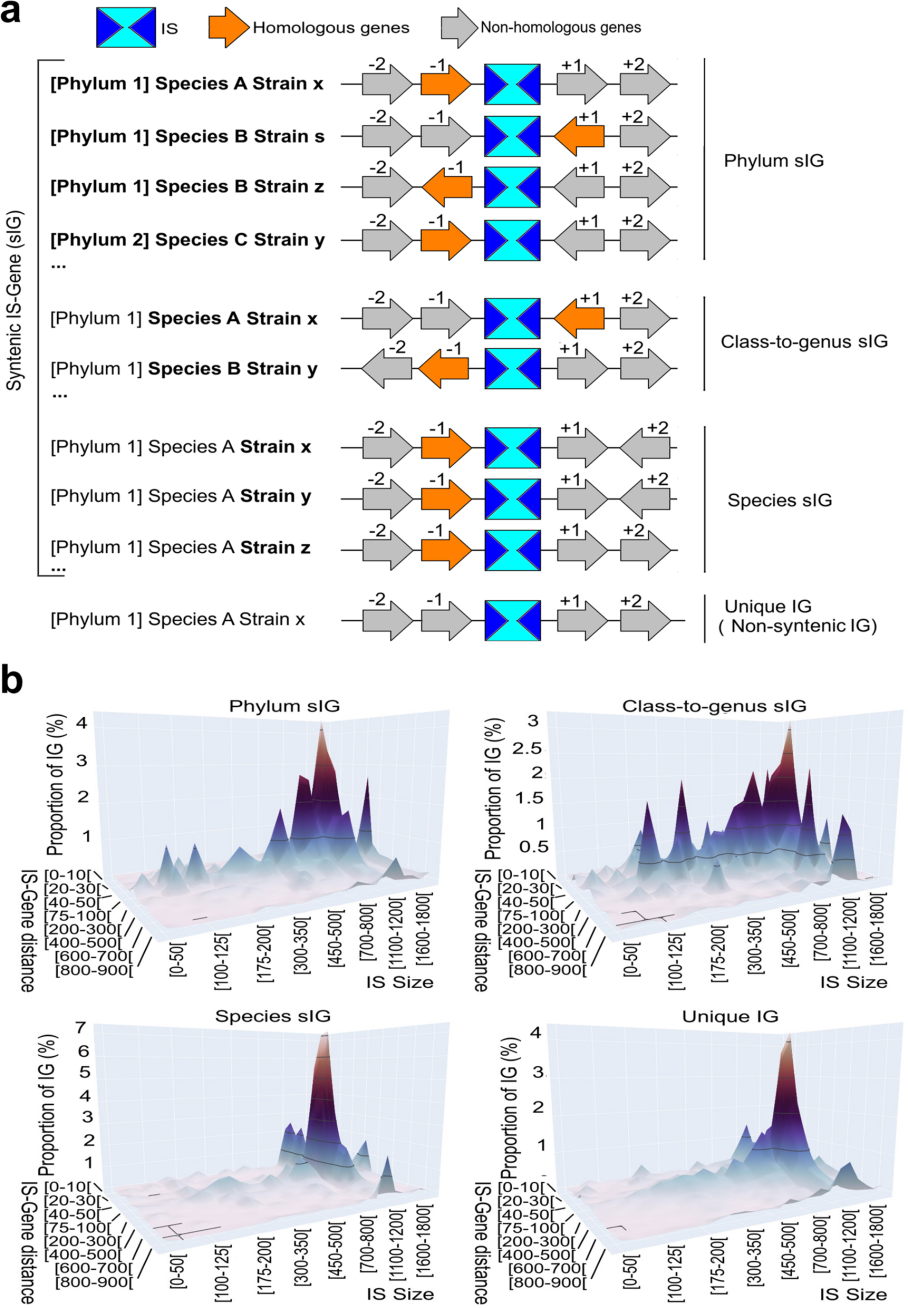
**a**. Rationale of syntenic IS-Gene (sIG) pairs. IS belongs to the same IS family. Colored genes correspond to homologous genes. Gray arrows are unique genes (without homolog). Phylum sIG, when IG couples are located in at least two distinct prokarotic phyla (Phylum 1 and Phylum2); Class-to-genus sIG, when IG belong to the same phylum but in distinct species, and therefore distinct taxonomic class, order, family or genus; Species sIG, when IG couples are only locate in one species but in distinct strains; and Unique IG, when IG couple is specific to one strain. Displayed configurations are given as examples. **b**. IS-Gene distance over IS size in syntenic IG pairs. 3D graphs display the proportion of IGs for size and IS-gene distance combinations in phylum, class-to-genus, and species sIGs and unique IGs. [50–100[means that the counting includes 50 but excludes 100

Using the concept of syntenic IS-Genes (sIGs) (Fig. 5a), comparative analysis toward taxonomic levels provided insights into the IS invasion mechanisms (including IS conservation), as well as arguments for the functional roles of ISs among prokaryotic genomes. We focused on ←IS→ shapes (104,644 IS occurrences in total), in which IS could play a role as a promoter in downstream and/or upstream neighboring genes. A significant BLAST E-value threshold was first defined through the exploration of the number of sIGs as a function of the E-values. Intersection between Phylum sIG, Species sIG and Unique IG curves (approximately 1e-50) were considered as the threshold E-value for the remaining study (Additional file 2), leading to 28,952 (27.7% of the total), 19,393 (18.5%), 28,363 (27.1%), and 27,936 (26.7%) IS occurrences for Phylum, Class-to-genus, and Species sIGs and Unique IGs, respectively (Additional file 3: Tables S10, S11, S12; Additional file 3: Tables S13, S14). However, only 8825 distinct ISs (8.4% of the 28,952 IS occurrences) participated in the formation of phylum sIGs, while among species sIGs and unique IGs, these proportions were 20.4 and 26.7%, respectively. It should be noted that the absence of phylum sIG sets with IS*Lre2*, IS*H3* and IS*H6* families, because these ISs are mostly located in one phylum (Firmicutes or Stenosarchaea). IS*5*, IS*21*, IS*4*, IS*607*, IS*91*, IS*L3* and IS*200* displayed two to four times more ISs in phylum and/or class-to-genus sIGs compared to the other IS families. This result indicates that these seven families were probably inserted earlier than others and were then subjected to horizontal transfer or IS transposition events and therefore conserved through evolution due to their positive roles in hosts.

### Short IS-gene distances reflect the role of ISs on gene expression

Except for IS*Lre2* and IS*Kra4* with a preferential insertion in the [50–100[and [100–250[bp distance classes, an overview of the distance distribution between ISs and closest genes showed that the number of IS occurrences increased with the lowest distance between ISs and neighboring genes (Additional file 5: Table S15). As an example, IS*6* of Firmicutes exhibited 24.6, 14.9 and 11.6% of the overall ISs of the phylum for the [1–50[, [50–100[and [100–150[bp distance classes, respectively. At the taxonomic level, the gene distance distribution seems closely related to the phylum. On the contrary, in Beta- and Gammaproteobacteria phyla, IS*1595* and IS*607* have a preferential insertion for the [500–550[bp and [300–350[bp distance classes to the closest genes, respectively. Next, IS-Gene distance and IS size distributions of sIGs were also explored for each sIG category (Fig. 5b; Additional file 5: Tables S16, S17). The four sIG categories displayed the same highest peaks for the [75–100[bp IG distance interval with numerous ranges of IS size for phylum and class-to-genus sIGs and ~ 1200 bp (lengths of most reference IS sequences) for species sIG and unique IG (Fig. 5b). These results confirm the hypothesis that species sIG and unique IG belong to recent IS insertions in host genomes. Moreover, the large variation in IS length from unique IGs to phylum sIGs, combined with similar IS-Gene distances, also suggest that IS length is the main factor changing over evolution, while the distance between an IS and the neighboring gene remains constant. In particular, 10,782 and 7407 ISs were located in phylum and class-to-genus sIGs, respectively, with IS-Gene distances less than 100 bp. Among them, 947 (8.8% of the total) and 912 ISs (12.3% of the total) were found in phylum and class-to-genus IG couples less than 10 bp from the neighboring gene, respectively; and 3834 (35.5% of the total) and 1039 (14.0% of the total) ISs overlapped the 5’UTR of the neighboring gene, respectively. As examples, IS*21* (120 IS occurrences), IS6 (75) and IS*L3* (111), showed phylum sIG sets with ISs within the proximal promoter (less than 50 bp). Moreover, the average distances between ISs and the neighboring gene within ←IS→ shapes were ~ 236 bp (i.e., ~ 118 bp for each ‘promoter’ region upstream of the gene). As previously described [23], these results confirm on a large scale that IS occurrences are often inserted in promoter regions with IS-Gene distances less than 100 bp and thus, IS would interfere with or drive the expression of proximal genes.

### The Betaproteobacteria, Crenarchaeota, Calditrichae, Planctomycetes, Acidithiobacillia and Cyanobacteria phyla act as IS reservoirs for other phyla

Network analysis of the connected phyla was limited to phylum sIG pairs to make the resulting network graphs easily understandable, with the size of the node (or circle, here the phylum) corresponding to the number of IS in the phylum and the edges the number of phylum sIG sets between two connected phyla (Additional file 5: Table S18; Additional file 6). For most of the IS families, Gamma-, Alpha-, and Betaproteobacteria, Firmicutes and Actinobacteria phyla display larger nodes and therefore suggest that these highly connected phyla may act as IS reservoirs for other prokaryotic phyla. However, when normalizing the data by the number of IS-containing genomes in the phyla, it appears that the reservoirs could be Betaproteobacteria, Crenarchaeota, Calditrichae, Planctomycetes, Acidithiobacillia and Cyanobacteria/Melainabacteria phyla with up to 159 ISs per genome. For each IS family, the main network properties at each node, including the degree of the node (which qualitatively represents the number of interactions (links) with other phyla), the weight of the node (or measure of how strong a particular interaction (link) is [here, the number of phylum sIG pairs among the two phyla]) and the strength of the node, which is the sum of the weights (the total number of phylum sIG pairs attached to links (interconnected phyla) belonging to a phylum), were explored (Additional file 5: Table S18). Indeed, the IS*200*/IS*605*, IS*21*, IS*3*, IS*5* and IS*L3* networks displayed the highest number of interconnected links between phyla, with up to 23 interconnections for Actinobacteria with other phyla. The highest strengths of interconnected phyla were observed for IS*110*, IS*200*/IS*605*, IS*3*, IS*4*, IS*481*, and IS*5,* with up to 1706 phyla sIG pairs within the IS5 network and up to 185 phylum sIG pairs shared between Gammaproteobacteria and Betaproteobacteria. When combining the strength and degree of the nodes for each IS family, IS*3*, IS*4*, IS*5* and IS*200*/IS*605* retained the strengthened and mostly connected networks with specific and major phyla, including Gamma-, Alpha-, and Betaproteobacteria, Firmicutes, Actinobacteria, Cyanobacteria, and Stenosarchaea. Altogether, the IS family networks of phylum sIG pairs displayed a large variety of network shapes but allowed us to highlight the following evidence: (*i*) phyla with few IS occurrences are linked together, therefore suggesting that IG couples (at least the IS occurrences) can be horizontally transferred in new phyla – that was the case for the IS982 network, for which the Deinococcus-thermus phylum is linked only to the Cyanobacteria phylum that is itself only linked to the Firmicutes phylum and (*ii*) distantly related phyla such as Cyanobacteria, Stenosarchaea and Firmicutes can share ISs through their genetic contexts, as seen with the phylum sIG pairs. All these observations indicate specific and preferential attachment of ISs with some phyla and therefore could imply positive roles of ISs in those phyla.

### Neighboring gene functions in phylum syntenic IGs are mostly related to transcriptional regulators

Since Phylum sIG displays strong and evolutionary conserved links between IG, our analysis was focused on phylum sIG, which results to: (*i*) ~ 68.5% of the phylum sIG sets show at least three phyla sIGs with up to 17 distinct phyla (Additional file 7: Table S19); (*ii*) the IS*H3*, IS*H6* and IS*Lre2* families do not have phylum sIGs, while the IS*21*, IS*5*, IS*91*, and IS*L3* families exhibit the highest numbers of distinct phyla in a phylum sIG set with 17 (from 1178 ‘IS*21* – ATP binding protein’ IG couples in which ‘ATP binding protein’ is the main function), 15 (from 298 ‘IS*5* – methyl-accepting chemotaxis protein’ IG couples), 15 (from 245 ‘IS*91* – site-specific tyrosine recombinase XerD’ IG couples) and 14 (from 368 ‘IS*L3* – restriction endonuclease subunit S’ IG couples) distinct phyla, respectively. These four phylum sIG sets involved 157 to 456 genomes from major phyla (e.g., Betaproteobacteria, Cyanobacteria/Melainabacteria group, and Actinobacteria), suggesting evolutionary links that exist between ISs and their associated neighboring genes. A large variety of functions were found associated with phylum sIGs, with major functions in phylum sets being related to ‘MFS transporter’ (57 sets), ‘ABC transporter ATP-binding protein’ (41 sets) and ‘LysR family transcriptional regulator’ (34 sets). These functions are also associated with distinct IS families. As examples, ‘MFS transporter’ function (in 772 sIG couples) and ‘LysR family transcriptional regulator’ (in 579 sIG couples) were associated with 17 [IS*1*, IS*110*, IS*1380*, IS*1595*, IS*200*, IS*21*, IS*256*, IS*3*, IS*30*, IS*4*, IS*481*, IS*5*, IS*607*, IS*630*, IS*66*, IS*982*, and IS*L3*] and 14 [IS*1*, IS*110*, IS*200*, IS*21*, IS*256*, IS*3*, IS*4*, IS*481*, IS*5*, IS*6*, IS*630*, IS*66*, IS*L3*, and IS*NCY*] IS families, respectively. This observation suggests a close relationship between ISs and genes involved in biological transcriptional processes.

**Fig. 6:**
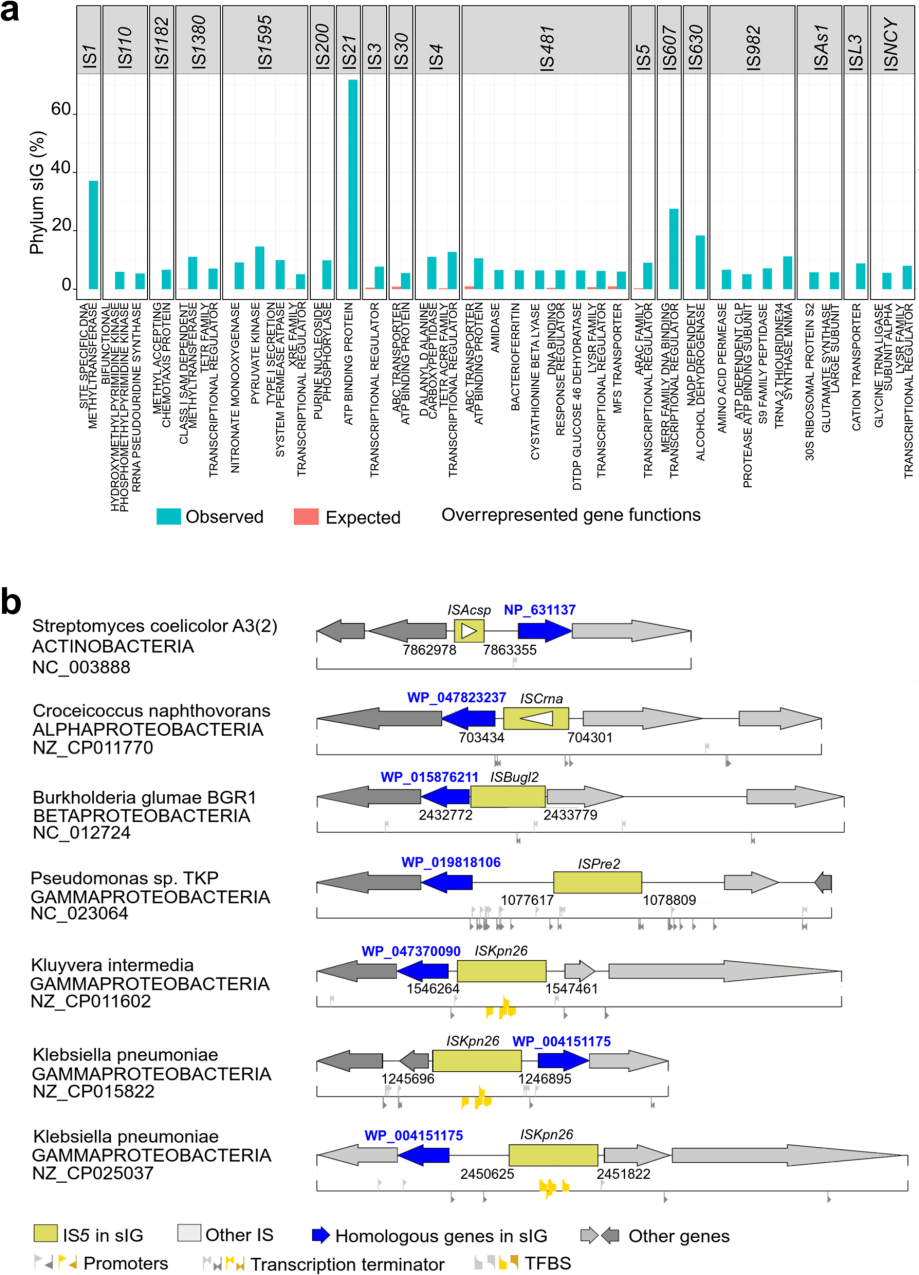
**a**. Overrepresented neighboring gene functions in the sIG pairs. sIG pairs were classified as overrepresented (or underrepresented) if the observed number was 10% greater than the expected value (See Materials and methods). Graphs display the overrepresented gene functions with observed proportions greater than 5% and more than 50 IG couples. The blue (red) bar is the observed (expected) proportions under a random distribution. **b**. A typical example of IS5 – ‘DNA-binding response regulator’ sIG pair and its genetic context. For each IG couple, the first line displays the ISs (rectangles) and the genes (arrows), while the second line shows the predicted (in gray) or experimentally known (in gold) regulatory motifs, including TFBSs, promoters and transcription terminators. Blue genes and the yellow IS family (here IS5) are involved in the syntenic IG (sIG) pair. For each IG couple, the genome name, phylum name, NCBI accession number, and coordinates of the genetic environment as well as the IS name and gene name of the IS-Gene participating in the sIG pair are shown

To determine whether the above observations on phylum sIGs were significant, statistical analysis was performed, resulting in 399, 26, and 896 phylum sIG sets that exhibited normal, under- and overrepresented distributions, respectively (Additional file 7: Table S20). Except for ‘hypothetical protein’ and ‘transposase/integrase/recombinase’ gene functions, selection of overrepresented sIG sets (containing at least 50 IG couples each, with at least 5% of the total sIG sets) led to 36 phylum sIG sets with a diversity of functions (pyruvate kinase, amidase, amino acid permease, etc.) and spanning 18 distinct IS families (Fig. 6a). The three most important overrepresented sIGs, ‘IS*21* - ATP Binding Protein,’ ‘IS*1* - Site specific DNA methyltransferase,’ and ‘IS*607* - MERR family DNA binding transcriptional regulator’, accounted for 71.9, 37.2 and 27.6% of the total IG couples of the IS families, respectively (Fig. 6a). In addition, 9 over 36 overrepresented sIG sets exhibited “transcription regulator” as closest gene functions. Moreover, gene functions such as LysR and TetR transcriptional regulators were both associated with two distinct IS families, IS*NCY* and IS*41* and the IS*4* and IS*1380* families, respectively. These results also suggest that IS sequences targeted ‘transcriptional regulator’ group of genes at all taxonomic levels, from phylum to genus (see also Fig. 4a), even if specific regulators (e.g., LUXR transcriptional regulator) exhibit ‘normal’ or underrepresented associations with IS families. Transcriptional regulators are known as ‘helix-turn-helix’ genes and function like transcriptional repressors or antibiotic regulators (e.g., TetR) [28–30]. Since these regulators and ISs are all subjected to horizontal transfer through various mechanisms such as transformation, transduction and non-canonical mechanisms involving membrane vesicles, nanotubes or phage-like gene transfer agents [28, 31], this could explain the widespread presence of IS sequences in many phyla. Therefore, the overrepresentation of IS-‘transcriptional regulator gene’ sIGs and their conservation over phylum lineages clearly suggests that the IS sequence plays a positive regulatory role, such as a promoter, when this couple enters a prophage in a new host genome.

### Deciphering the regulatory role of ISs on neighboring genes

Among the database regulatory motifs (see Materials and methods), 57,205 (predicted promoters and transcription terminators accounted for 94.7 and 0.4%, respectively; and 4.9% experimentally known as TFBSs) were located in IS occurrences of the ←IS→ shapes (Additional file 8: Tables S21, S22, S23). These predicted and experimentally known regulatory motifs were located within 27,768 distinct ISs, representing an average ratio of ~ 2 motifs per motif-containing IS. Knowing that within the ←IS→ shapes the role of ISs as promoters becomes crucial to promote gene transcription in the forward and/or reverse orientations (in particular when the IS-Gene distance is less than 100 bp), our results consolidate our hypothesis about the regulatory role of IS occurrences in ←IS→ shapes.

At the IS level, ~ 26.5% of ISs were found to contain regulatory motifs, among which more than 98% were predicted motifs. Indeed, experimental regulatory motifs were often found within the IS*5* (1950), IS*1* (495) and IS*3* (297) families, representing up to 64.4% of the overall motifs within the IS family. Similarly, motif-containing ISs were mostly found in IS*481* (35.5% of total IS occurrences with motifs; 9841 ISs in total), IS*5* (13.0%) and IS*3* (12.9%) families, while no motif (experimental or predicted) was observed in IS*H3* and IS*H6*. To our knowledge, the observed low numbers of regulatory overlapping motifs (compared to the number of IS sequences) in some IS families (e.g., IS*Azo13*, IS*91* and IS*Kra4* families) are probably due to the low number of experimental data available (~ 100 genomes contain experimental motifs among the 9037 genomes). Most of the IS families exhibited the highest proportions of sIGs with overlapping motifs within the species sIG category (e.g., IS*110*, IS*701* and IS*5*), with up to 65.6% (Additional file 8: Tables S21, S22 and S23). However, the highest amounts of motif-containing IG were observed for phylum or class-to-genus sIG categories. Therefore, conserved motif-containing ISs in phylum sIG category clearly suggest the importance of regulatory motifs located in IS for the expression of proximal genes.

Next, manual cross-checking and validation of these data were performed using experimentally published IG couples from the Vandecraen review [9]. Indeed, the authors described 40 ISs with a complete outward-directed promoter and 28 ISs with outward-directed − 35 promoter components, which displays the putative regulatory roles of ISs for the neighboring genes. Twenty-four of the 68 ISs (from [9]) were found in our results, and 7 of them were located in the same class-to-genus sIG set (e.g., IS*21* – ‘class A beta-lactamase’ IG present in both *Bordetella holmesii* F627 and *Bacteroides fragilis*). Note that missing regulatory motifs within these experimental regulatory ISs may be due to changes within gene names and/or organism names between the Vandecraen paper and NCBI website (e.g., blaCTX-M-2 in NCBI vs. blaCTX-M2, CTX-M, B4U25_43495, and DM059_36235 within the paper and UniProt database). While these facts constitute limitations of the IS regulatory roles, it clearly remains one of the ways to extract useful information that provides clues about the putative regulatory roles of ISs. Among the phylum sIG category, the IS*5* – ‘DNA-binding response regulator’ phylum sIG set possessed 31 IG couples (7 are shown) spread across six phyla (4 are shown) (Fig. 6b). Except for the IS occurrences of Streptomyces, other IS sequences harbor at least one regulatory motif (including promoters and transcription terminators), and three (one in *K. intermedia* and two in *K. pneumoniae*) of them have experimental regulatory motifs. Moreover, the three IG couples in *K. intermedia* and both in *K. pneumoniae* present similar and experimentally known TFBSs in both orientations of the IS sequence, thus confirming their functional role as enhancers for the DNA-binding response regulator gene [15–17]. The IG couples in *Pseudomonas* sp. TKP and *Burkholderia glumae* BGR1 predict transcription terminator motifs, but their IS-Gene distance (33 bp) is too small to create a promoter without the IS sequence. Consequently, this IS could be a promoter for the DNA-binding response regulator gene. The IG couple in *Croceicoccus naphthovorans* presents two promoter motifs within the downstream gene, with one inside the IS sequence, that could be an alternative promoter for the gene. All these observations strongly suggest a potential regulatory role of ISs (as promoters, TFBSs or transcription terminators) through their associations with neighboring genes.

## Discussion

IS-containing organisms live in changing environments and/or with genetic exchanges, therefore allowing ISs to transfer from one genome to another [4, 32]. Recently, the distribution and phylogenetic relationships of IS*6* members, their impact on their host genomes as well as transposition pathways was reviewed [33], therefore demonstrating the importance of an IS family in generating clusters of clinically important antibiotic resistance genes [1]. To tackle the functional and regulatory roles of ISs on proximal genes, a large-scale genomic identification of IS occurrence as well as the introduction of IS-Gene (IG) concept were performed. Then, syntenic IG (sIG) comparative analysis over the prokaryotic lineages was investigated with IS-Gene distances and functions of the neighboring genes.

While in silico, the IS identification procedure used restrictive BLAST parameters, thus providing good specificity for the identified ISs. Indeed, IS occurrences were identified, even if they were complete or incomplete (e.g., IS fossil) but with a minimal size of 80 bp and 80% sequence identity to ensure the specificity of detection. Our IS identification strategy emphasized that shorter IS occurrences (with 80–200 bp size) result from (*i*) complete IS occurrence ancestors that were fragmented or subjected to deletion and/or mutation during the evolutionary time and for which the host genome has conserved useful sequence fragments compared to the entire IS sequence and/or (*ii*) the blast procedure itself, which basically finds regions of local similarity between sequences leading to shorter sequences. However, the use of BLAST does not introduce identification bias since most of the IS occurrences (in 20 IS families) displayed similar lengths (i.e., with +/− 20% difference) when compared to the IS reference sequence sizes. Note that manual curation of these IS occurrences, as currently done by ISfinder [20], would be impossible for all 9037 genomes. In addition, combined results of IS network analysis (which highlighted a number of phyla as IS reservoirs) and the IS family distribution among the phyla clearly suggest that IS spreading remains influence by host multiple factors (as mentioned above) and not by the histories of species.

Using the concept of the IS-Gene (IG) couple, we first demonstrated in a large scale that almost all identified ISs are located less than 500 bp from the closest gene regardless of the host genome. It was shown that for IG distances greater than 500 bp, IS copies appear highly and rapidly mutated or deleted, probably due to the fast evolution rate observed for ISs in prokaryotes [1]. Our results clearly suggest that IS insertions, when too close or inside a neighboring gene, generally decrease the fitness of the host genome, and when too far to neighboring genes, the host genome will remove the ‘useless’ sequences through recombination and other evolution mechanisms. Thus, ISs should be inserted in a good distance range conserved long enough, form an IS-Gene couple within genomes, and finally play an IS regulatory role. As suggested for Archaea [34], IS insertions relative to the gene orientations were not randomly distributed in our study, since the proportions of IG shapes (←IS→, →IS←, →IS→ and ←IS←) remained variable and the patterns of under- and overrepresentation of the insertions were found specific for one or a set of IS families. Overrepresentation of the →IS← shapes would mean that the IS insertion in the 3′ or intergenic region does not disturb gene regulation. In contrast, the underrepresentation observed for ←IS→ shapes mean that IS insertions in the promoter region disturb gene regulation and consequently decrease host fitness.

Our findings also demonstrated that IS conservation at its insertion site relies on their distance to neighboring genes, as well as the corresponding gene functions. The IG gene functions highlighted major functions (e.g., ATP binding protein and MFS transporter) that were conserved over distantly related phyla, therefore significantly suggesting their putative IS roles. However, functions with synonyms or written differently may have introduced some bias. As an example, IS*1634* is associated 18 times with the ‘glycosyl transferase’ function and 8 times with the ‘glycosyltransferase’ function. Fortunately, the identification of orthologous genes with BLAST partially removes synonym bias problems, but this approach needs to define an E-value threshold (here 1e-50), which could also introduce variation in IS-Gene couples. Note that in some cases, ‘IS - hypothetical protein’ couples were mainly found because ~ 20% of the prokaryotic genes do not have a clearly defined function. Statistical analysis showed that gene functions related to ‘transcriptional regulators’ are overrepresented in close proximity of many IS family. The high diversity of gene functions associated with IS may suggest the following hypothesis: ISs are randomly inserted in the host genome (regardless of gene function), and they are conserved if the IS lead to a positive or silent role. Therefore, we emphasize that a genome environment or a specific function alone could not be sufficient for widespread IGs in genomes. It should be noticed that we cannot distinguish insertion events that only transfer IS sequences from those that might include other genes (case of IS-Gene couples). In the latter, the IS sequences would serve as vectors to spread the neighboring genes. In contrast, when a gene plays an essential role (e.g., transcription factor) in most organisms, its association with an IS would create a ‘mobile promoter’ [11–13] used for HGT, which will be subsequently spread over the taxonomic lineage. Several studies have shown that the most beneficial role of IS insertion remains the creation of a new promoter for neighboring genes [11–13, 35, 36]. Moreover, it was suggested that MITEs are often found close to or within genes and are involved in gene regulation [37]. We demonstrate on a large genomic scale that this process is not specific to a set of organisms but occurs in all prokaryotic lineages, and therefore provides information about ISs that could use the closest gene as promoter.

Altogether, key gene players (transcriptional regulators, transporters, and ATP binding protein) related to adaptation to particular environments may be significantly widespread through the IG mobile vehicle and therefore contribute to the formation of syntenic IGs (sIGs), as they increase host fitness or have a silent role over taxonomic lineages. In this context, comparative genome analysis of sIGs revealed that more than 26% of ISs contained regulatory motifs in phylum or class-to-genus sIGs. However, these observations are underestimated due to the lack of chipSeq data. Indeed, there are a limited number of genomes (~ 100 genomes) with experimental chipSeq data, most of which are concentrated in a few model genomes (~ 10). In addition, even without chipSeq data, the location of ISs within the ←IS→ shapes (for which the average gene distance is ~ 236 bp) suggests that ISs must drive the regulation of one or both neighboring genes. As an example, IS-Gene distances within the ‘IS*21* - HAMP domain-containing protein’ (from *Aeromonas veronii*) and the ‘IS6 - cation:proton antiporter’ (*Pyrococcus furiosus* COM1) are 10 and 19 bp, respectively, suggesting that the full promoter size of each gene must contain a part of the IS occurrence. All these results consolidate the regulatory role of ISs in ←IS→ shapes, in which ISs become crucial as promoters to fulfil gene transcription in forward and/or reverse orientations, in particular with short IS-Gene distances.

**Fig. 7:**
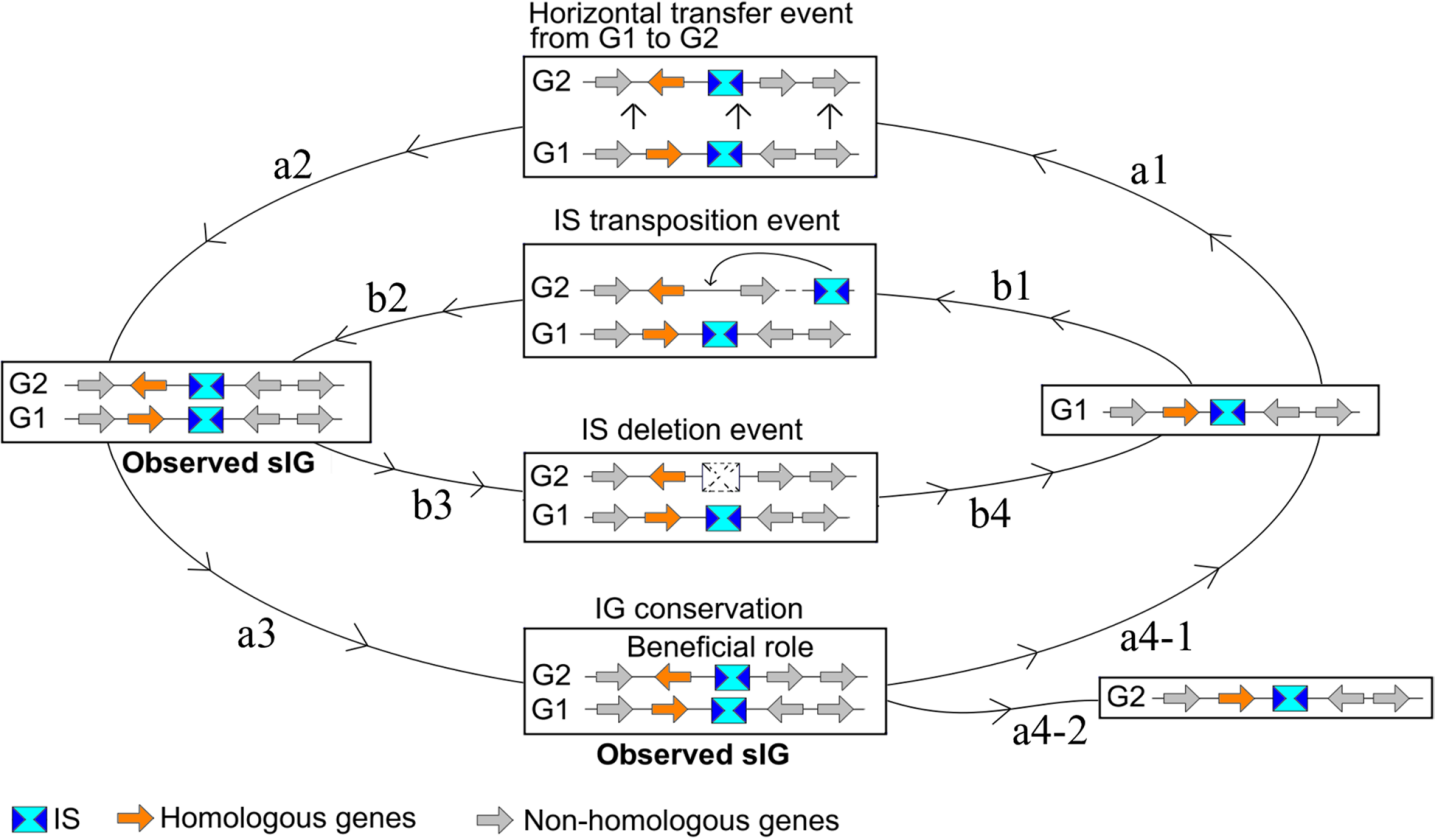
Evolutionary model of the IS-Gene couples. The arrows and rectangles correspond to genes and IS occurrences, respectively. The colored arrows are homologous genes. From the G1 block (set of genes including the IS and homologous and nonhomologous genes) (here G1), horizontal gene transfers (steps a1 and a2) and IS transposition events (steps b1 and b2) lead to the creation of a new block (here G2) in another genome. An IS deletion event can yield a unique (or nonsyntenic) IG (steps b3 and b4). During evolution, a given IS can be conserved through the evolutionary history leading to an observed syntenic IG (sIG) when an IS plays a positive role (i.e., regulatory motifs for neighboring genes) in the host genome (step a3). G1 or G2 blocks return to the starting point for new evolutionary events (steps a4–1 and a4–2)

Unique IG and sIG categories were explained with an IS-Gene evolutionary model (Fig. 7). Indeed, from a given G1 block (set of genes including the IS, homologous and/or nonhomologous genes), horizontal gene transfer and/or IS transposition events (steps a1 and b1) could lead to the creation of the G2 block in another organism (steps a2 and b2) and therefore an observed sIG. These sIG results were as follows: (*i*) species sIGs for ISs that were recently inserted in close ancestor strains, followed by vertical inheritance, and finally remained specifically conserved in different strains among the species and/or (*ii*) phyla sIGs or class-to-genus sIGs with ISs that were either inserted early in an ancestor or novel species before speciation and/or horizontal gene transfers of the sIG blocks into phyla or classes, orders, families and genera. In the next evolutionary step, sIGs could delete ISs (or the neighboring gene) (step b3) within one of the G blocks (here G2) if the IS has a neutral/negative role on the neighboring gene, leading to the extinction of the sIG block and reformation (step b4) of a unique IG. The latter ‘unique IG’ could also represent ISs that were inserted recently in a new host location. During the evolutionary process, a sIG could also be conserved due to its beneficial role in the host genome (step a3). The positive roles of ISs in the regulation of neighboring genes were extensively studied. As examples, IS*903* (IS5 family) and IS*981* (IS*3* family) activate downstream genes in *Paracoccus* spp. and the ldhB gene in *Lactococcus lactis,* respectively, through regulatory motifs (i.e., promoter signal) located in their internal sequence [18, 19, 38]. Finally, each of the G blocks could also undergo (steps a4–1 and a4–2) the three evolutionary events described before.

## Conclusion

In a large-scale genomic analysis, we identified IS occurrences in prokaryotic genomes, then we defined IS-Gene (IG) couple and syntenic IG concepts in order to decipher functional and evolutionary relationships between IS families and neighboring genes. The main findings are: (*i*) IS-neighboring gene functions are mainly related to transcriptional and transport activities but with transcriptional regulators in the case of phylum syntenic IGs; (*ii*) short IS-Gene distance highlights putative roles of IS on neighboring gene expression; (iii) cross-comparisons of IS occurrences with known and predicted regulatory motifs lead to ~ 2 motifs per motif-containing IS, which in combination with the ←IS→ shapes, clearly consolidate the regulatory role of IS on the neighboring genes. The precise regulatory role of IS on the neighboring genes, however, requires further investigations. Our findings demonstrated that IS conservation at its insertion sites relies on their distance to neighboring genes and the corresponding gene functions, and for which an evolutionary model was provided. Our study also establishes a solid foundation for further investigations for a specific IS in any particular prokaryotic organism.

## Materials and methods

### Genome, insertion sequence (IS) and regulatory sequence data

Genome data and IS reference data were downloaded from the NCBI ftp database (ftp.ncbi.nlm.nih.gov/refseq/release/) and ISfinder (www-is.biotoul.fr), respectively, in February 2018 (see Additional file 1: Table S1; Additional file 8: Table S24). The genome data included 8786 and 251 genome (chromosomes and plasmids) sequences from bacteria and archaea, respectively, together with their annotation features. For a better understanding, ‘genome’ was denoted as a set of genomic data sharing the same NCBI assembly identifier (e.g., GCA_000832965.1 for *Bacillus anthracis*) and ‘species’ was related to a set of genomes that had two identical first words in their organism names without strain identifiers (e.g., *Escherichia coli*). The term ‘genus’ was used for the set of species that had the same first name in the NCBI genome name (i.e., *Escherichia*), and the terms ‘phylum,’ ‘class,’ ‘order,’ and ‘family’ were used as in the NCBI taxonomy lineage, except for with proteobacterial classes. It should be noted that due to the high number of genomes within Proteobacteria classes (e.g., Alphaproteobacteria), those classes were considered and analyzed as phylum taxonomic clades in this paper.

IS reference data included 4628 known IS members (nucleotide and protein sequences) grouped into 29 families (Additional file 8: Table S24) [20]. IS subgroups were not considered in our study since more than 50% of the IS members do not have a defined subgroup in the ISfinder database.

Regulatory sequences such as transcription factor binding sites (TFBS), riboswitch motifs, promoters and transcription terminators (as defined by [39]) were provided from the following sources: (*i*) experimental regulatory databases (containing manually curated knowledge from peer-reviewed publications) including CollectTF [40], RegulonDB [41], DBTBS [42], Prodoric2 [43], and RegTransBase [44]; (*ii*) a predicted regulatory motif database (without manually curation), Genome2D [39]; and (*iii*) the literature [45–47].

### Identification and distribution of IS occurrences and IS-gene (IG) couples

IS member (nucleotide and protein) sequences were aligned against the 9037 genomes using the Needleman-Wunsch algorithm from BLASTn and tBLASTn (BLAST Suite 2.6.0) [48] (ftp.ncbi.nlm.nih.gov/blast/executables/blast+/2.6.0), respectively. Then, *PERL* scripts were written to identify IS occurrences as follows: Blast hit alignment regions of the genomes were first subjected to the 80:80 rule (> 80 bp alignment size with at least 80% sequence identity) [49]. In the case where several IS member sequences are located in the same hit region, only those with the best E-value were selected for further analysis. Contiguous IS occurrences that belong to the same family, with less than an 80 bp gap sequence between them, were merged to form an IS occurrence. A total list of IS occurrences is shown in Additional files 9 and 10. Preliminary analysis also showed that (*i*) 16 IS families have members with identical sequences (i.e., 100% BLAST identity) and (*ii*) the other 13 IS families exhibit IS members with at least 80% sequence identity (data not shown). Consequently, most of the raw BLAST hits from IS member sequences belonging to the same IS family overlap the same genome regions. This preliminary analysis demonstrated that IS members from the same family exhibit very similar sequences and could subsequently be considered homologous. Therefore, subsequent IS analysis was performed at the IS family level.

IS distributions among complete genomes were mainly performed relative to their presence/absence in each genome, taxonomic clade, and IS family, as well as their host organism lifestyles and IS features, including IS size and distance between the IS and neighboring genes. All identified IS occurrences are described in Additional files 9 and 10. IS hotspots were defined as genomic locations that contained at least three consecutive ISs, with nucleotide distances between two ISs lower than the cumulative sequence lengths of both ISs and without any annotated genes. For each IS, the annotation features of the two closest genes (i.e., noncoding genetic objects are not considered here) were extracted to define the IS-Gene shapes. Details are in Fig. 3a.

### Analysis of neighboring gene orientations and gene functions in IG

For each type of IG shape (Fig. 3b) and for each IS family (and for the overall IS), a *𝛘*^*2*^ test was applied by comparing the number of observed and expected IG shapes under a normal distribution (i.e., 25% of each IG shape). When the *𝛘*^*2*^ test showed a statistical bias (*p value* < 0.01), IS families were classified as overrepresented (or underrepresented) for an IG shape if the observed value was 10% greater (or lower) than the expected value.

Gene functions were extracted from the ‘product’ field of the GenBank files. Two genes were considered to have identical functional names if identical characters were found, including uppercase and lowercase letters. From the 117,851 distinct function names, those for which the number of identical gene names was equal to or greater than 3072 (representing 0.01% of the overall number of genes in all analyzed genomes) were first selected, leading to approximately 1759 distinct gene function names. In the next step, ‘IS - GeneFunction’ (IGF) couples were defined as the association of an IS occurrence and neighboring gene functions. Therefore, each IS sequence leads to two or three ‘IS - GeneFunction’ couples from the relative position − 1 to + 1 and depends on whether there is an overlap between the IS and an existing gene.

### Syntenic IS-gene couples (sIG) analysis

Two genes were considered homologous if there was a BLAST alignment between the two genes with an E-value lower than a given threshold (irrespective of their gene function names). Two IG couples were defined as a ‘presyntenic IS-Gene’ (presIG) if the following criteria were observed: (*i*) the two ISs of the IG belonged to the same family and (*ii*) at least two of the four neighboring genes were homologs and located within two distinct taxonomic clades or two distinct species. Several presIGs were grouped together as a syntenic IS-Gene (sIG) set depending on the taxonomic levels (the priority order was phylum, class/order/family/genus, and species); into phylum sIG and class-to-genus sIG sets when the taxonomic levels were first related to phylum and class/order/family/genus, respectively; and finally into species sIG sets if two IS-Gene couples were only located in distinct strains but from a unique species (see Fig. 4a). Therefore, a syntenic IG sets may have at least two distinct taxonomic clades and can harbor multiple IGs from the same taxonomic clade. In addition, an IS-Gene couple located in only one strain (i.e., without homologous gene) was considered a non-syntenic IG couple and called a ‘unique IG’ couple. Note that the neighboring gene function analysis in sIG was performed as for IG. For regulatory motif analyses, the location of IS occurrences was cross-compared (using in-house scripts) with the location of gene regulatory sequences (TFBS, promoter regions, transcription terminators, and riboswitches; see above), leading to overlapping regulatory motifs within IS sequences.

### Statistical analysis

Statistical tests on IS distribution in and along the genomes, neighboring gene functions and orientations, and overlapping regulatory motif analyses were performed using R [50].

#### IS distribution in prokaryotic genomes

A uniform IS distribution corresponds to the number of IS-containing genomes within phyla when IS are randomly inserted in these genomes. *Student’s t tests* were used to determine whether the IS insertions had a nonuniform distribution.

#### Neighboring gene orientations in IG

For each type of IG shape and for each IS family (and for the overall ISs), a *𝛘*^*2*^ test was applied by comparing the number of expected (25% of the overall observed IG under a normal or random distribution) and the four observed IG shapes. When the *𝛘*^*2*^ test showed a statistical bias (*p value* < 0.01), IS families were then classified as overrepresented (or underrepresented) for an IG shape if the observed value was 10% greater (or lower) than the expected value.

#### Neighboring gene functions in IGs

Gene function distributions relative to IS occurrence in IGs were calculated, and the observed gene function number was compared with the expected number under the normal distribution (ISs are inserted randomly close to gene functions) using the *𝛘*^*2*^ test (*p value* threshold of 0.01).

#### Other statistical analyses

Except for the IS distribution in prokaryotes that uses the *Student’s t test* and the IS hotspots that use the *Wilcoxon-Mann-Whitney* test to observe the uniform distribution of the IS along the genome sequence, the *𝛘*^*2*^ statistical test was used. The *𝛘*^*2*^ test highlights the difference between the observed and expected distributions if the ISs were randomly inserted (called ‘normal’ or ‘uniform’ distribution). Statistical tests were only applied on genomes containing at least 10 ISs, and the statistical threshold to determine whether the observed distribution was a normal distribution was set to 1% (i.e., 1% or a *p value* < 0.01). Statistical analysis of neighboring gene functions was limited to IGF couples for which the gene function name represents at least 0.01% (3072 annotations) of all annotated genes.

#### IS-hotspots distribution and analysis

A *Wilcoxon-Mann-Whitney* statistical test was first used to detect whether the IS occurrence distribution along genomes displays a nonuniform distribution (i.e., unequal distribution of IS locations along the genome sequence). Indeed, we emphasize that in a uniform distribution (i.e., random insertion of ISs within genome sequences), IS hotspots have fewer chances to appear (or to exist). If a genome displays a nonuniform IS distribution, a *PERL* script was used to analyze the IS locations relative to the gene locations and to identify IS hotspots based on the criteria defined above.

## Supplementary Information

**Additional file 1.**

**Additional file 2.**

**Additional file 3.**

**Additional file 4.**

**Additional file 5.**

**Additional file 6.**

**Additional file 7.**

**Additional file 8.**

**Additional file 9.**

**Additional file 10.**

## Data Availability

All data generated or analysed during this study are included in this published article. The accession numbers of genome datasets used during the current study are provided in Additional file 1: Table S1, column A.

## Abbreviations

HGT: Horizontal gene transfer
IS: Insertion sequence
IG: A Couple forms by an IS and its neighboring gene
IGF: A couple forms by an IS and the neighboring gene function
sIG: Syntenic IG
TFBS: Transcription factor binding sites
UTR: Unstranslated region
