# Supplementary figures and images for: From a large-scale genomic analysis of insertion sequences to insights into their regulatory roles in prokaryotes
Source: BMC Genomics. 2022 Jun 20;23:451. doi: 10.1186/s12864-022-08678-3 (PMC9208149; doi:10.1186/s12864-022-08678-3)

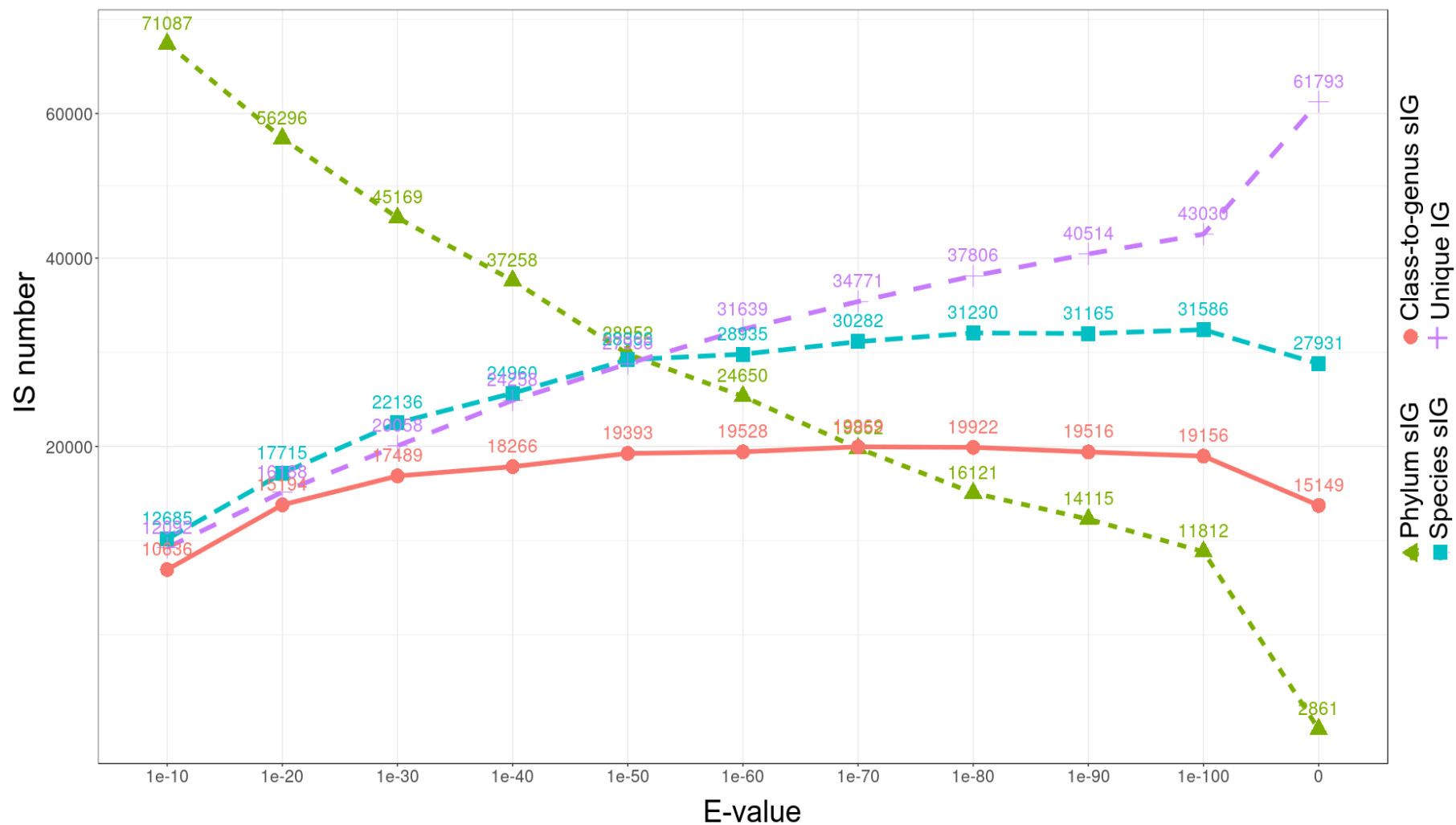

**Additional file 2.** Number of IS-Gene couples in function of Blast E-value thresholds.

Supplement: Supplementary file 2 — Additional file 2. [file 12864_2022_8678_MOESM2_ESM.pdf]
