# Supplementary material for: From a large-scale genomic analysis of insertion sequences to insights into their regulatory roles in prokaryotes
Source: BMC Genomics. 2022 Jun 20;23:451. doi: 10.1186/s12864-022-08678-3 (PMC9208149; doi:10.1186/s12864-022-08678-3)
Supplement: Supplementary file 6 — Additional file 6. [file 12864_2022_8678_MOESM6_ESM.pdf]

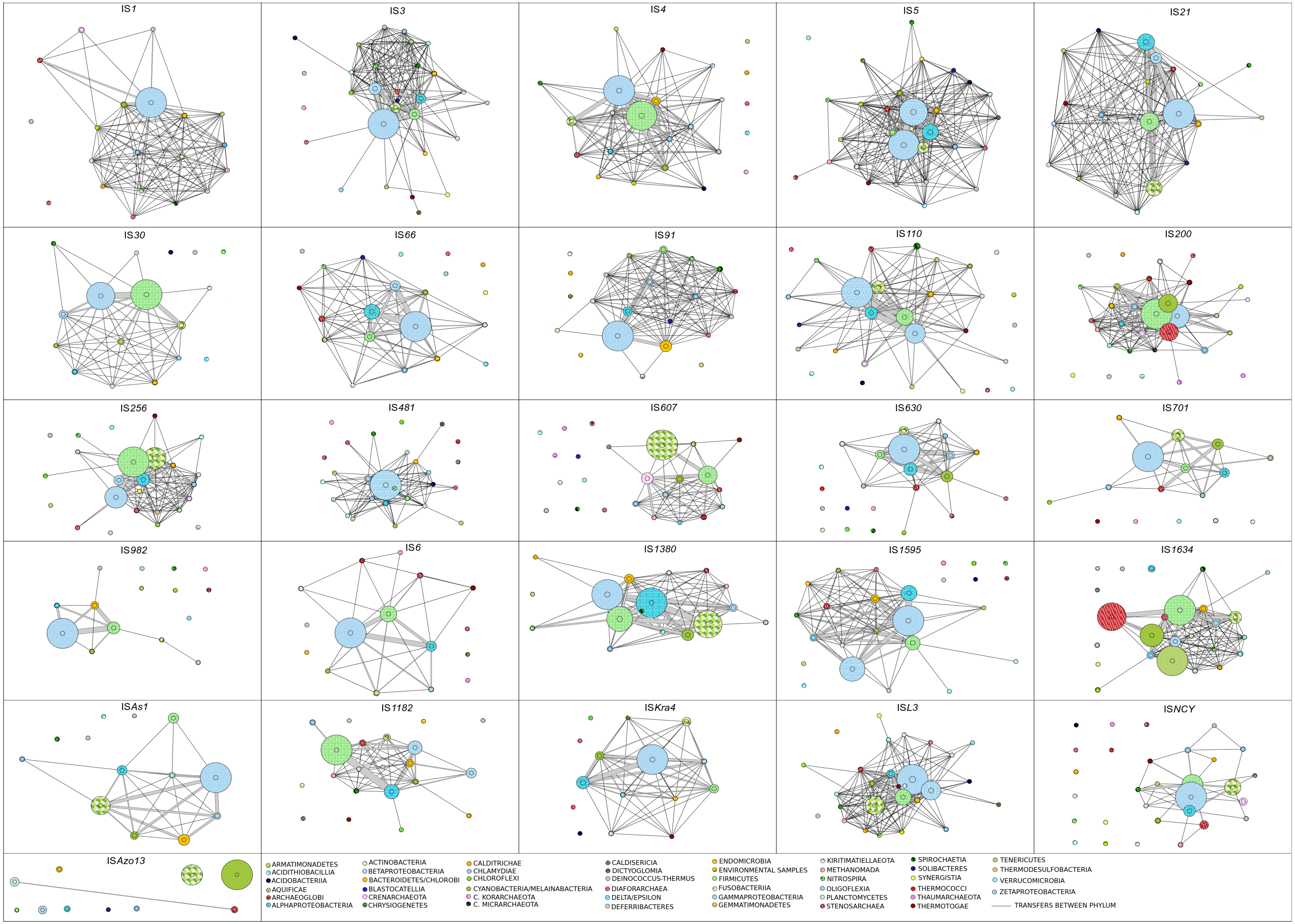

Additional file 6. Phylum sIG networks.
For each Is family, the nodes (or circles) correspond to the number of IS in the phylum and the edges correspond to the number phylum sIG sets between two connected phyla. Each graph was drawn with its own scale.
